# A study of positioning orientation effect on segmentation accuracy using convolutional neural networks for rectal cancer

**DOI:** 10.1002/acm2.12494

**Published:** 2018-11-12

**Authors:** Kuo Men, Pamela Boimel, James Janopaul‐Naylor, Chingyun Cheng, Haoyu Zhong, Mi Huang, Huaizhi Geng, Yong Fan, John P. Plastaras, Edgar Ben‐Josef, Ying Xiao

**Affiliations:** ^1^ University of Pennsylvania Philadelphia PA USA

**Keywords:** convolutional neural networks, deep learning, positioning orientation, rectal cancer radiotherapy, segmentation

## Abstract

**Purpose:**

Convolutional neural networks (CNN) have greatly improved medical image segmentation. A robust model requires training data can represent the entire dataset. One of the differing characteristics comes from variability in patient positioning (prone or supine) for radiotherapy. In this study, we investigated the effect of position orientation on segmentation using CNN.

**Methods:**

Data of 100 patients (50 in supine and 50 in prone) with rectal cancer were collected for this study. We designed three sets of experiments for comparison: (a) segmentation using the model trained with data from the same orientation; (b) segmentation using the model trained with data from the opposite orientation; (c) segmentation using the model trained with data from both orientations. We performed fivefold cross‐validation. The performance was evaluated on segmentation of the clinical target volume (CTV), bladder, and femurs with Dice similarity coefficient (DSC) and Hausdorff distance (HD).

**Results:**

Compared with models trained on cases positioned in the same orientation, the models trained with cases positioned in the opposite orientation performed significantly worse (*P *<* *0.05) on CTV and bladder segmentation, but had comparable accuracy for femurs (*P *>* *0.05). The average DSC values were 0.74 vs 0.84, 0.85 vs 0.88, and 0.91 vs 0.91 for CTV, bladder, and femurs, respectively. The corresponding HD values (mm) were 16.6 vs 14.6, 8.4 vs 8.1, and 6.3 vs 6.3, respectively. The models trained with data from both orientations have comparable accuracy (*P *>* *0.05), with average DSC of 0.84, 0.88, and 0.91 and HD of 14.4, 8.1, and 6.3, respectively.

**Conclusions:**

Orientation affects the accuracy for CTV and bladder, but has negligible effect on the femurs. The model trained from data combining both orientations performs as well as a model trained with data from the same orientation for all the organs. These observations can offer guidance on the choice of training data for accurate segmentation.

## 1 INTRODUCTION

Segmentation of the organs‐at‐risk (OARs) and the tumor target is one of the key problems in the field of radiotherapy. Computer‐assisted automated methods have the potential to reduce the inter‐ and intra‐observer variability and relieve physicians from the labor‐intensive contouring workload. Such problems have been addressed in clinical applications using “atlas‐based” automated segmentation software.^1^, ^2^, ^3^ Despite the popularity of such software, the recent deep learning revolution, especially the fully convolutional neural networks (CNN),^4^, ^5^, ^6^, ^7^, ^8^ has turned the tables due to its significant improvement in terms of segmentation accuracy, consistency, and efficiency. Lustberg et al.^9^ and Lavdas et al.^10^ demonstrated that CNN contouring demonstrated promising results in CT and MR image segmentation as compared with atlas‐based methods. Ibragimov et al.^11^ successfully applied CNN for OAR segmentation in the head and neck CT images. The authors^12^ previously reported a dilated CNN with high accuracy for segmentation of rectal cancer. With the promising learning tools and the enhancement of computer hardware, deep learning will dramatically change the landscape of radiotherapy contouring.^13^

As is well‐known, data are one of the most important components of any machine learning system,^14^ especially for the deep networks.^15^, ^16^ Although the approaches substantially improve the performance, training CNN requires a large number of fine quality contour annotations to achieve a satisfactory segmentation outcome. The training data for modeling must be representative of the characteristics of the image sets in the study. Special attention should be paid to collecting and constructing an appropriate dataset for any segmentation system for CNN. Patients undergoing radiotherapy for rectal cancer are generally treated either in a prone position to reduce the volume of small bowel in the high‐dose region^17^ or in a supine position as it is much more stable.^18^ A different positioning orientation (prone or supine) will result in variability^19^ in location, shape, and volume of the structures of interest. These differences may affect segmentation performance when training and testing across different positioning orientations.

In this study, we investigated the effect of cross‐orientation on segmentation for rectal cancer radiotherapy using CNN. This issue is highly relevant for the following reasons. First, whether a CNN model trained with patients positioned in one orientation performs poorly for cases in the opposite orientation has not been studied before. Although this may be subjectively true, there have been no experiments to support this assumption and no quantitative evaluation of such deterioration. Second, there has been no prior report on whether and how much the training with data from both orientations would affect the segmentation accuracy. More data can increase the diversity, but mixing two very different types of data are likely to lead to confusion in model training. This is an open question whose answers may influence the training strategies of deep learning. Third, segmentation is often the prerequisite of medical image analysis. If the positioning orientation affects segmentation, it will also affect further quantitative analysis, e.g., radiomics, which is based on the segmentation. This study will therefore provide evidence and guidance for patients positioning orientation considerations.

## 2 MATERIALS AND METHODS

### 2.A Patients data and pre‐processing

Planning CT Data of 100 patients with rectal cancer who underwent radiotherapy were collected for this study. Half of them (50 patients) were acquired in supine position and the remaining 50 patients were positioned prone. CT images were reconstructed with a slice thickness of 3 mm. Because the pixel sizes of two‐dimensional (2D) CT slices were not uniform among different cases, they were all resampled to a matrix of 512 × 512 with a resolution of 1 × 1 mm^2^. It was performed using MATLAB's “imresize” function with bi‐cubic interpolation and anti‐aliasing. A total of 8831 and 8846 CT slices were taken for the supine and prone datasets, respectively. Physicians contoured the CTV and OARs on the 2D CT images as part of the clinical care. We chose three regions of interest (ROIs) to evaluate the effect of positioning orientation on segmentation: clinical target volume (CTV), bladder, and femurs.

The image data were pre‐processed in MATLAB R2017b (MathWorks, Inc., Natick, MA, USA). A custom‐built script was used to extract and label all the voxels that belonged to the specific contours from the DICOM structure files. We used a contrast‐limited adaptive histogram equalization (CLAHE)^12^, ^20^ algorithm to pre‐process the CT images for image enhancement. For the patients in the “supine” position, the images were rotated 180° clockwise to create the corresponding “virtual prone” images. This is to remove the effects that are entirely caused by the physical orientation of the image. The final data used for CNN were the 2D CT slices and the corresponding 2D labels. The process and the additional image pre‐processing were fully automated.

### 2.B Convolutional neural networks implementation

**Figure 1:**
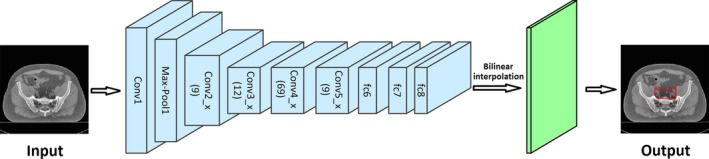
The procedure of segmentation using CNN.

We used the ResNet‐101^7^ as the deep learning network for segmentation. As illustrated in Fig. 1, the inputs of the network were the original 2D CT slices and the outputs were the corresponding maps with the segmentation labels. Table 1 shows the detailed architecture of ResNet‐101. It has 101 parameter layers and has become one of the state‐of‐the‐art methods for segmentation due to its high accuracy and efficiency. Similar to all other CNN, ResNet‐101 is composed of the convolutional layers, the max‐pooling layer, and the fully convolutional (fc) layer. The convolutional layers extract features from the input image. There is a batch‐normalized (BN) operation following each convolutional layer. An additional operation called Rectified Linear Unit (ReLU) is used to introduce non‐linearity in CNNs by replacing all negative pixel values in the feature map by zero, i.e., output = max (0, input). The most important feature of ResNet is the residual connection which is inherently necessary for training very deep convolutional models. The residual connections add skipped connections that bypass a few convolutional layers at a time. Each bypass gives rise to a residual block in which the convolutional layers predicted a residual that is added to the input tensor of the block.

**Table 1:** The detailed architecture of ResNet‐101

Layers	Shape				
	Kernel	Padding	Stride	Channel	Output
Input data	**–**	**–**	**–**	**–**	M×N
Conv1	[7 × 7]	3	2	64	M/2 × N/2 × 64
Max‐pool1	[3 × 3]	1	2	64	M/4 × N/4 × 64
Conv2_x	$\left[\begin{array}{c} 1\times 1\\ 3\times 3\\ 1\times 1 \end{array}\right]$× 3	$\left[\begin{array}{c} 0\\ 1\\ 0 \end{array}\right]$	$\left[\begin{array}{c} 1\\ 1\\ 1 \end{array}\right]$	$\left[\begin{array}{c} 64\\ 64\\ 256 \end{array}\right]$	M/4 × N/4 × 64 M/4 × N/4 × 64 M/4 × N/4 × 256
Conv3_x	$\left[\begin{array}{c} 1\times 1\\ 3\times 3\\ 1\times 1 \end{array}\right]$ × 4	$\left[\begin{array}{c} 0\\ 1\\ 0 \end{array}\right]$	$\left[\begin{array}{c} 2\\ 1\\ 1 \end{array}\right]$	$\left[\begin{array}{c} 128\\ 128\\ 512 \end{array}\right]$	M/8 × N/8 × 128 M/8 × N/8 × 128 M/8 × N/8 × 512
Conv4_x	$\left[\begin{array}{c} 1\times 1\\ 3\times 3\\ 1\times 1 \end{array}\right]$ × 23	$\left[\begin{array}{c} 0\\ 2\\ 1 \end{array}\right]$	$\left[\begin{array}{c} 1\\ 1\\ 1 \end{array}\right]$	$\left[\begin{array}{c} 256\\ 256\\ 1024 \end{array}\right]$	M/8 × N/8 × 256 M/8 × N/8 × 256 M/8 × N/8 × 1024
Conv5_x	$\left[\begin{array}{c} 1\times 1\\ 3\times 3\\ 1\times 1 \end{array}\right]$× 3	$\left[\begin{array}{c} 0\\ 4\\ 0 \end{array}\right]$	$\left[\begin{array}{c} 1\\ 1\\ 1 \end{array}\right]$	$\left[\begin{array}{c} 512\\ 512\\ 2048 \end{array}\right]$	M/8 × N/8 × 512 M/8 × N/8 × 512 M/8 × N/8 × 2048
fc6	[3 × 3]	1	1	2048	M/8 × N/8 × 2048
fc7	[1 × 1]	0	1	1024	M/8 × N/8 × 1024
fc8	[1 × 1]	0	1	2	M/8 × N/8 × 2
Interpolation	Factor = 8				M×N × 2
Output data					M×N

In detail, the input images have a size of M × N. Conv1 includes one convolutional layer that had a filter size of 7 × 7, a stride of 2, and a padding of 3. It generates 64 feature maps of size M/2 × N/2. A max‐pooling operation followed reduced the size of the feature image to M/4 × N/4. It can reduce the number of parameters in the network, and hence control overfitting. Conv2_x includes 9 (3 × 3) convolutional layers all generating feature maps of size M/4 × N/4. Conv3_x includes 12 (3 × 4) convolutional layers and generated feature maps of size M/8 × N/8 due to a max‐pooling operation in conv3_1. Conv4_x and conv5_x have 69 (3 × 23) and 9 (3 × 3) convolutional layers, respectively and the size of all the feature maps is M/8 × N/8. In the layers of fc6, fc7, and fc8, the fully connected layers are replaced with fully convolutional layers. The final 1 × 1 convolution in fc8 combines all the features non‐linearly and can predict pixel‐wise segmentation in images. The size of the input is reduced to one‐eighth of the original size in the fc8 layer due to the max‐pooling and stride operations. Therefore, a bilinear‐interpolation is applied after fc8 to recover the original size as the final output.

## 3 EXPERIMENTS

In order to evaluate the effect of positioning orientation on segmentation, we designed the following three sets of experiments for comparison.

1. Segmentation using the model trained with data from the same orientation;
2. Segmentation using the model trained with data from the opposite orientation;
3. Segmentation using the model trained with data from both orientations.

We performed fivefold cross‐validation for evaluation. For each loop of validation, 80% of the data were used as the training set to “tune” the parameters of the segmentation model, and the remaining 20% cases were used as the test set to evaluate the performance of the model. In detail, the datasets of supine and prone were randomly divided into five equal‐sized subsets (supine subsets: S_j_, j = 1, 2, 3, 4, 5; prone subsets: P_j_, j = 1, 2, 3, 4, 5), respectively. First, we trained the first set of models: Model_S_1_ (training set: S_2_, S_3_, S_4_, and S_5_), Model_P_1_ (training set: P_2_, P_3_, P_4_, and P_5_), and Model_SP_1_ (training set: S_2_, S_3_, S_4_, S_5_, P_2_, P_3_, P_4_, and P_5_). We performed six scenarios of test with these three models, respectively:

1. Segmentation on S_1_ using Model_S_1_;
2. Segmentation on S_1_ using Model_P_1_;
3. Segmentation on S_1_ using Model_SP_1_;
4. Segmentation on P_1_ using Model_P_1_;
5. Segmentation on P_1_ using Model_S_1_;
6. Segmentation on P_1_ using Model_SP_1_.

Subsequently, we chose subsets with j as the testing sets and i != j as the training set to train the *j*th set of models. We repeated this step until we trained five sets of models to cover all the data.

In order to avoid overfitting during training phase, we adopt an offline and online data augmentation schemes. The offline augmentation randomly transformed the training cases with noise pollution and rotation (between −5° and 5°), which enlarged the training dataset by ten times. The online augmentation applied methods of randomly scaling the input images (from 0.5 to 1.5), randomly cropping, and randomly left‐right flipping. With the data augmentation, the network hardly trained the same augmented image twice, as the modifications were performed at random each time. This greatly increased the diversity of samples and made the net more robust.

We implemented the training and testing of our model using Caffe,^21^ which is a publicly available deep learning platform. The model was trained in a 2D pattern. During the testing phase, all the 2D CT slices were tested one by one. In detail, the 2D CT slices were the inputs and the corresponding segmentation probability maps were the outputs. The model parameters for each network were initialized using the weights from the corresponding model trained on ImageNet.^22^ In this case, the input channel of “Conv1” layer should be three. However, our input was the gray image of CT, which has only one channel. We solved this problem by taking only the first channel of each filter in the “Conv1” pre‐trained on ImageNet when loading the model. This was achieved by modifying the original code of Caffe, that is, to compare the channel number c1 of the current network and the channel number c2 of the pre‐training model. If c1 is less than c2, only previous c1 channel of the filters is used. The training set was used to “tune” the parameters of the networks. The loss function and the training accuracy were computed with “SoftmaxWithLoss” and “SegAccuracy” built‐in Caffe, respectively.^21^ The optimization algorithm of training used backpropagation with the stochastic gradient descent (SGD). We used the “poly” learning rate policy where current learning rate equals the base one multiplying ${\left(1-\frac{\mathrm{iter}}{\max\_iter}\right)}^{\mathrm{power}}$. In this study, we set the base learning rate to 0.001 and power to 0.9. The batch size was set to 1 due to the limitation of physical memory on GPU card. The training iteration number was set to 90K. The momentum and weight decay were set to 0.9 and 0.0005, respectively. The training and testing phases were fully automated with no manual interaction. All computations were undertaken on an Amazon Elastic Compute Cloud with NVIDIA K80 GPU.

### 3.B Performance evaluation

Physician approved manual segmentation was used as the gold standard reference. The spatial consistency between the automated segmentation and the manual reference segmentation was quantified using two metrics: the Dice similarity coefficient (DSC)^23^ and the Hausdorff distance (HD).^24^ Because the image segmentation was performed in 2D mode, we calculated the two metrics slice by slice and then took the average as the final results for each patient. The value of DSC ranges from 0, indicating no spatial overlap between the two segmentations, to 1, indicating complete overlap. The HD indicates the degree of mismatch between the two segmentations. Smaller value usually represents better segmentation accuracy. Paired Student's t‐tests were used to determine whether there were significant differences between different train‐test scenarios. IBM SPSS Statistics software (version 24.0; IBM Inc., Armonk, NY, USA) was used for all statistical analyses. *P* < 0.05 was considered significant.

## 4 RESULTS

The results of the segmentation accuracy are summarized in Tables 2 and 3. The CNN segmentation models for CTV and bladder trained with cases positioned in the opposite orientation performed significantly worse (*P* < 0.05) than that trained with cases positioned in the same orientation, with average DSC values of 0.74 vs 0.84 and 0.85 vs 0.88 and HDsmm of 16.6 vs 14.6 and 8.4 vs 8.1 for CTV and bladder, respectively. In particular, the average DSC values were 0.07 (CTV supine: 0.83–0.76), 0.12 (CTV prone: 0.84–0.72), 0.03 (bladder supine: 0.88–0.85), and 0.03 (bladder prone: 0.88–0.85) lower (*P* < 0.05), respectively. The corresponding HDsmm were also significantly larger (*P* < 0.05) with average values of 0.8 (CTV supine: 15.7–14.9), 3.2 (CTV prone: 17.4–14.2), 0.2 (bladder supine: 8.5–8.3), and 0.5 (bladder prone: 8.3–7.8), respectively. However, segmentation of the femurs with both models showed high and comparable accuracy (DSC: 0.91 ± 0.02 vs 0.91 ± 0.02, *P* > 0.05; HD: 6.3 ± 1.5 vs 6.3 ± 1.6, *P* > 0.05).

**Table 2:** DSC results for different types of train‐test combinations

Train on	CTV		Bladder		Femurs	
	Test on		Test on		Test on	
	Supine	Prone	Supine	Prone	Supine	Prone
Supine	0.83 ± 0.04	0.72 ± 0.07	0.88 ± 0.05	0.85 ± 0.06	0.91 ± 0.02	0.90 ± 0.03
Prone	0.76 ± 0.06	0.84 ± 0.04	0.85 ± 0.08	0.88 ± 0.04	0.91 ± 0.02	0.90 ± 0.03
Both	0.83 ± 0.04	0.84 ± 0.04	0.88 ± 0.06	0.88 ± 0.05	0.91 ± 0.02	0.90 ± 0.02

**Table 3:** Hausdorff distance (mm) results for different types of train‐test combinations

Train on	CTV		Bladder		Femurs	
	Test on		Test on		Test on	
	Supine	Prone	Supine	Prone	Supine	Prone
Supine	14.9 ± 2.8	17.4 ± 3.0	8.3 ± 2.8	8.3 ± 1.8	6.3 ± 1.3	6.3 ± 1.6
Prone	15.7 ± 2.8	14.2 ± 2.6	8.5 ± 2.7	7.8 ± 2.0	6.3 ± 1.5	6.3 ± 1.7
Both	14.7 ± 2.8	14.1 ± 2.4	8.4 ± 2.8	7.7 ± 1.9	6.4 ± 1.5	6.2 ± 1.5

As for the models trained with data from both orientations, their segmentation accuracy was as good as models trained on data from the same orientation for all the three organs. They have almost the same average DSC of 0.84 vs 0.84, 0.88 vs 0.88, and 0.91 vs 0.91 and HDs of 14.4 vs 14.6, 8.1 vs 8.1, and 6.3 vs 6.3, respectively. No significant difference was found (*P* > 0.05) between the two types of train‐test combinations.

**Figure 2:**
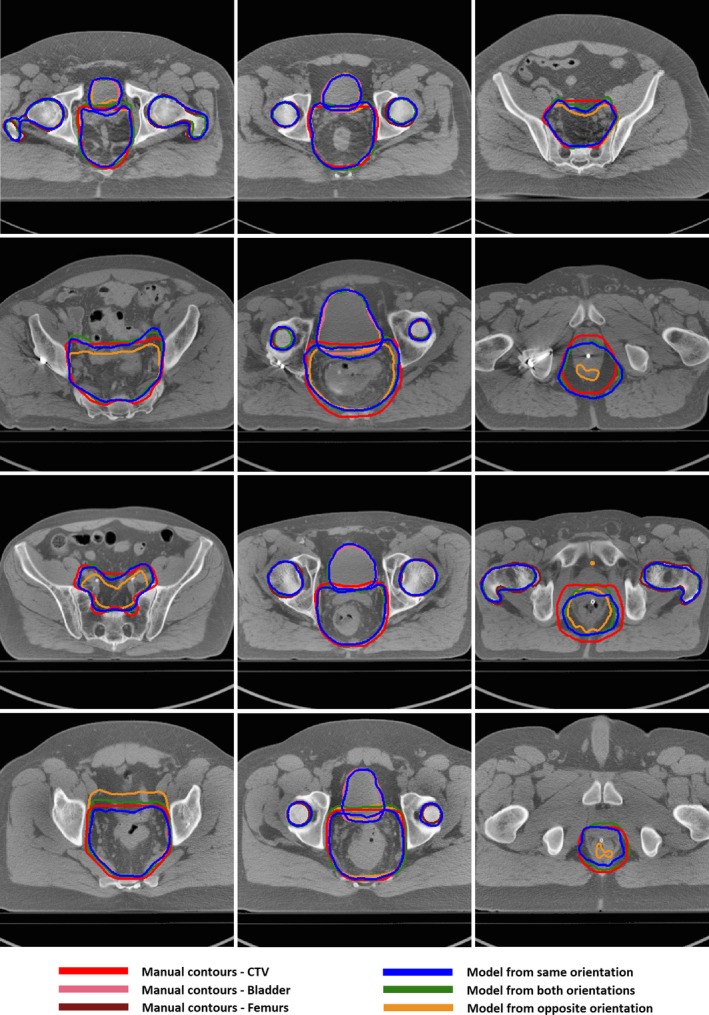
Segmentation results on cases position in supine using CNN models trained with different types of datasets.

**Figure 3:**
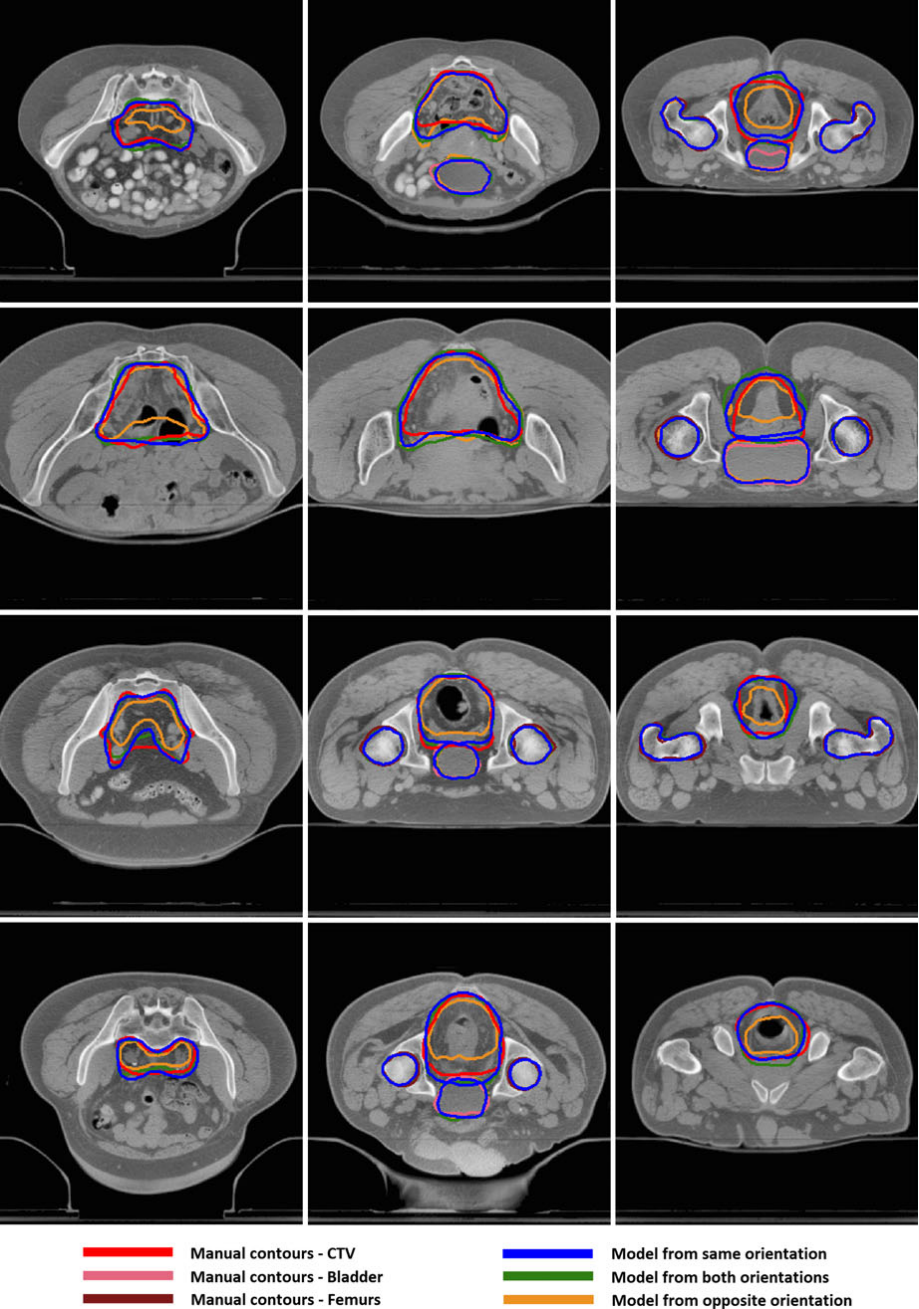
Segmentation results on cases positioned in prone using CNN models trained with different types of datasets.

Figures 2 and 3 illustrate results in different orientation. Compared with models trained with cases positioned in the opposite orientation, the auto‐segmented contours with models trained with data from the same or both orientations were in better agreement with the manual contours for CTV and bladder, but similar for femurs.

## 5 DISCUSSION

In order to further confirm the result is independent of the chosen network, we also performed the corresponding experiment with U‐Net^25^, which is used widely for medical segmentation. The average DSC values with U‐Net models trained with cases positioned in the same, opposite, and both orientations were (0.82, 0.75 and 0.82) for CTV, (0.86, 0.82 and 0.87) for bladder, and (0.89, 0.89 and 0.89) for femurs, respectively. Although the overall result was inferior to ResNet‐101, the positioning effect on segmentation demonstrated a consistent trend.

To the best of our knowledge, this is the first study that investigates the effect of positioning orientation on segmentation for rectal cancer radiotherapy using CNN. The experiments demonstrate that the performance of the model does not depend on the orientation for segmentation of femurs; however, model trained on data from different orientation reduces the accuracy of CTV and bladder segmentation. The reasons for the inferior performance for CTV and bladder but comparable performance for femurs when trained with data from a different orientation can be explained as follows: Most of the deep learning methods work under the common assumption that the training data and the testing data have identical feature spaces with underlying distribution. However, soft tissue in the human pelvis can be greatly deformed with different orientations, which will lead to significant differences in morphology, location, range, and other aspects of the CTV and bladder. Differences in such features inevitably affect segmentation performance when training and testing across different orientations. Unlike the CTV, the bladder has a clearer boundary under both orientations. CNN can extract and make use of the boundary features; therefore the effect of position orientation on the bladder is less obvious than the CTV. The femurs are not deformable and have clear boundaries with surrounding soft tissues. As a result, once the feature distribution of the testing data remains stable, the segmentation model can be used effectively on a cross‐dataset. This finding is a bit intuitive; however, many subjective hypotheses have been proven to be false in fact. We tested this hypothesis through a lot of experiments and finally proved it with quantitative analysis, which is very meaningful for clinical practice.

Another very important finding is that model trained on data combining both orientations works as well as model trained with data from the same orientation for all the organs. When we trained the model using data from both orientations, the training data can represent the entire scenario of testing data. CNN can capture the features of different orientations by fully discovering and exploiting regularities in the training data. These representative features can accurately be applied to the testing set. This may be the reason for the good performance of the model trained on data combining both orientations. This meaningful experiment provides support that we can train a universal model with patients of different orientations together, which can increase the stability of the model and reduce the complexity of training.

Recent breakthroughs in segmentation for radiotherapy have been mainly accelerated by the approaches based on CNN. It aims to increase levels of automation and replace very time‐consuming human interventions as well as improve accuracy and efficiency. Due to the “data‐hungry” nature of CNN and the difficulty of collecting large‐scale image data in radiotherapy applications, the performance is limited by relatively few segmentation datasets. In this study, we investigate the effect of cross‐orientation on segmentation and find that it does not influence the segmentation accuracy of bone. This means that we can use datasets with patients in different orientations to augment the amount of data, which will improve the accuracy and stability of CNN learning. Our experiments also demonstrated that segmentation accuracy of the soft tissue with deformation depends on the training data being consistent in positioning. We need to be very careful in selecting the training set to ensure that it represents the image characteristics accurately.

Segmentation is the first step of the image analysis, e.g., radiomics. Given that the orientation affects the features used for target segmentation, whether it makes a difference in radiomics features is worthy of research. The differences introduced in radiomics features may affect clinical outcome analysis.

In practical scenarios, we often have model learned with CNN on large datasets but would like to apply it to other cases that have significant differences in image statistics with limited training data. Collecting new labeled training data and forming a particular model could be time‐consuming and labor‐intensive. Combining CNN with transfer learning is a potential solution. Transfer learning allows the feature distributions used in training and testing to be different. It can transfer image representations acquired previously to new but similar tasks to solve problems faster and more effectively. Segmentation using CNN with transfer learning will be explored in the future.

## 6 CONCLUSIONS

The experiments demonstrated that the orientation of the training dataset affects the accuracy of CNN‐based segmentation for CTV and bladder but has negligible effect on the femurs. The model trained from data combining both orientations works as well as model trained on data from the same orientation for all the organs. These observations provide guidance on how to choose training data for accurate segmentation.

## CONFLICT OF INTEREST

No conflicts of interest.
